# Gene expression profile of a selection of Polycomb Group genes during zebrafish embryonic and germ line development

**DOI:** 10.1371/journal.pone.0200316

**Published:** 2018-07-09

**Authors:** Naomi D. Chrispijn, Karolina M. Andralojc, Charlotte Castenmiller, Leonie M. Kamminga

**Affiliations:** 1 Radboud University, Faculty of Science, Department of Molecular Biology, Radboud Institute for Molecular Life Sciences, Nijmegen, the Netherlands; 2 Radboud University Medical Center, Department of Molecular Biology, Nijmegen, the Netherlands; University Zürich, SWITZERLAND

## Abstract

Polycomb Group (PcG) genes are transcriptional repressors that are described to be important during development and differentiation. There is significant interest in PcGs proteins because of their role in stem cell biology and tumorigenesis. In this study we characterize the expression of a selection of PcG genes in the adult germline of zebrafish and during embryogenesis. In adults, expression of selected PcG genes is found to be enriched in germ line over somatic tissues. Therefore, the germ line of adult zebrafish was analyzed for the expression pattern of a selection of PcG genes by whole mount *in situ* hybridization. We detected presence of the tested PcG gene transcripts at early stages of both oogenesis and spermatogenesis. This enriched expression for early stages of gametogenesis is also observed in developing gonads at 4 and 5 weeks post fertilization. Additionally, zebrafish embryos were used to study the spatio-temporal expression patterns of a selection of PcG genes during development. The PcG genes that we tested are maternally loaded and ubiquitously expressed at early developmental stages, except of *ezh1*. The expression of the PcG genes that were assessed becomes enriched anteriorly and is more defined during tissue specification. The data shown here is an important resource for functional PcG gene studies *in vivo*.

## Introduction

Polycomb group (PcG) proteins are important negative epigenetic regulators of transcription by modifying histone tails [1]. PcG proteins were first identified for their role in maintaining cell identity, thereby determining patterning of *Drosophila* embryos [2]. In addition, PcG proteins are described to be involved in a plethora of other biological processes, which include differentiation, cell cycle control, X-chromosome inactivation, and tumorigenesis [3]. PcG proteins can assemble in so-called Polycomb Repressive Complexes (PRCs): PRC1 and PRC2. PRC2 consists of the core components Eed, Suz12, and Ezh1/2. The PcG proteins Ezh1 and Ezh2 are mutually exclusive and tri-methylate lysine 27 of histone H3 (H3K27me3). The core of PRC1 consists of Ring1A/B, a member of the Pcgf family, a Phc protein, and a Cbx protein. PRC1 is recruited to H3K27me3 and places the histone H2A lysine 119 ubiquitination mark (H2AK119Ub), which in turn stabilizes the repressive H3K27me3 mark [4–6]. PcG proteins and their associated molecular mechanisms by which they regulate transcription are evolutionarily conserved. The zebrafish genome has undergone genome duplication, however, over time some duplicated genes are lost or have taken a different function [5,7,8]. The high number of duplicates and even splice-variants of the different homologs can make gene function analysis a complex manner [5].

Regulation of gene transcription by PcG proteins and epigenetic gene regulation in general are dynamic processes that are shown to be important during development of multiple vertebrate systems. For example, in mice *Ezh2* and *Rnf2* were both found to be essential for embryonic development, since knock-outs were shown to be embryonic lethal [9,10]. This early lethality makes it challenging to study the function of *Ezh2* and *Rnf2* in the murine system [9,10]. In contrast, zebrafish PcG mutants are reported to survive gastrulation and serve as a very suitable model to study PcG function development [4,11–13]. Zygotic *rnf2* zebrafish mutants die around 4–5 days post fertilization (dpf), showing a range of phenotypes, including craniofacial defects, the absence of pectoral fins, and motility problems [13,14]. The zygotic *pcgf1* zebrafish mutant fish survive until adulthood, but display growth defects and premature aging [4]. Recently, *ezh2* zygotic zebrafish mutants were described to harbor intestinal problems and show lethality around 11 dpf [12]. The *ezh2* mRNA is maternally loaded and maternal zygotic *ezh2 (MZezh2)* mutants show defects in maintenance of cellular identity and tissue integrity and die around 2 dpf [11]. The different timing of lethality of zygotic *ezh2* mutants and *MZezh2* mutants indicates the importance of the maternal load for proper development.

Though PcG genes are known for their role in embryonic development, less is known about their role during gametogenesis. In germ cells, PcG genes are crucial for preservation of genetic integrity, generation of genetic diversity, and for transmission of genetic information to the progeny. In invertebrates such as *C*.*elegans* and *Drosphila*, PRC2 function has been studied during gametogenesis. In *C*.*elegans*, mutants for orthologues of PRC2, which are maternal sterile effect-2 and -6 (*mes-2* and *mes-6*), show that loss of maternal MES function leads to germ line degeneration and sterility [15,16]. *Drosophila* oocytes lacking *E(z*) (orthologue of Ezh2) were found to transdifferentiate into nurse cells [17]. This has led to the conclusion that cell fate relies on PRC2 functioning, and on the presence of the H3K27me3 mark in the germ line [17]. Recently, it was described that PRC2 plays a role in murine germ line development, as PRC2 aggregates in the nucleus of mouse fetal germ cells [18]. During murine germ line development DNA methylation is reduced, and H3K27me3 is the crucial repressive mechanism responsible for epigenetic reprogramming, which relies on Ezh2 functioning [18]. In addition, PcG proteins were suggested to be involved in vertebrate oogenesis by establishing developmental competence, since double knockout Ring1/Rnf2 murine oocytes show aberrant gene expression detected by changes in the maternal load [19].

Both male and female gametes are produced during the lifetime of zebrafish. This unique characteristic facilitates studying the different stages of oogenesis and spermatogenesis during adulthood [20]. During zebrafish development germ plasm is already present at the cleavage furrows of the 2-cell stage embryo. As from the 32-cell stage, germ plasm is found in distinct primordial germ cells, which have migrated to the start of the yolk extension by 1 dpf [21]. One of the genes that is highly expressed in these cells is the germ cell marker *vasa* [22]. Around 4 weeks post fertilization zebrafish sex is determined. Zebrafish oocytes contain maternal mRNA and are externally fertilized and the zygote undergoes rapid cell divisions. Around 3.3 hours post fertilization (hpf) mid-blastula transition (MBT) takes place. At MBT the zygotic genome is activated (ZGA) and the maternal mRNA load is degraded. Already before MBT epigenetic marks are reported to be present in zebrafish, albeit at very low levels [23]. Especially at ZGA epigenetic marks, including H3K27me3, show a dramatic increase at developmental regulatory genes [23].

To understand the role of the epigenome during embryonic and gonad development, a crucial first step is to enhance the knowledge about PcG gene expression. Current data available on PcG expression during zebrafish embryogenesis and gametogenesis is limited. A recent publication shows the expression levels measured by RNA-sequencing at 18 developmental time points from 1 cell to 5 dpf [24]. This data serves as a useful database, however lacks spatial information. In addition, the spatial information on the expression of PRC core-components is incomplete, and often not available of the same stages of development. The gene expression of *pcgf* family members (*pcgf* genes, and *bmi1a/-b*), *phc2*, and of *ezh1* and *ezh2* during early zebrafish development has already been partly reported [4,6,11,25]. However, spatio-temporal data on the remaining PcG genes during embryogenesis is lacking. Also, PcG gene expression in the germ line of zebrafish is not studied. Importantly, the presence of a specific RNA transcript can serve as a proxy for the presence in the maternal load. Nevertheless, this does not give information about the expression and potential role during gametogenesis. In this study, we give an overview of the spatio-temporal expression of some of the main PcG genes, which assemble in PRC1 and PRC2. Since zebrafish serve as a unique vertebrate model for studying PcG genes in early development, the expression patterns of PcG genes profiles described in this paper are very useful for follow-up studies using PcG mutants. Moreover, our results can be used to get a better insight in the potential role of PcG proteins in germ line development and embryogenesis.

## Materials and methods

### Zebrafish maintenance

Zebrafish (wildtype strains: AB/Albino/TL) were maintained in water of 27.5°C in a 14/10h light/dark cycle. The evening before spawning, one male and one female were placed into a tank with a divider and placed together the following morning. Spontaneous spawning occurred when the male and female were put together at the moment the light turned on. Embryos were collected and staged according to Kimmel *et al*. [26]. The zebrafish experiments described in this study were conducted according to the Dutch and European Union guidelines for the handling of laboratory animals. The experimental procedures carried out on zebrafish were reviewed and approved by the Utrecht University and Radboud University Animal Experiments Committee.

### RNA extraction and RT-PCR

Adult fish were euthanized with 2-phenoxyethanol (0.1% v/v). Three biological replicates of ovary, testes, tail muscle, and eye were obtained by dissection [27] and homogenized in TriZol (Ambion). The ZYMO RNA microprep kit was used to isolate RNA and treat the samples with DNAseI. cDNA was generated using oligod(T) primers and Superscript reverse transcriptase (Promega). RT-qPCR was run on a BioRad machine in three technical replicates and the housekeeping gene *rsp18* was used as reference gene. The genes and primers used for RT-qPCR are shown in Table 1. As a control a minus reverse transcriptase sample for each condition was tested.

**Table 1:** RT-qPCR primer sequences.

	sequence forward primer (5’– 3’)	sequence reverse primer (5’– 3’)
*ezh2*	AAATCGGAGAAGGGTCCTGT	TCTGTTGGAGCTGAACATGC
*eed*	ATCTGGTACATGCGCTTCTC	GGTGGTGCACTTTGCTTTAT
*suz12a*	GCATGACCACCAGAGATACC	CAGGGCTCTCCTCTATCTCC
*rnf2*	GGATGGAGTCAGCGAGATTG	TCTACATTGATGGGGCTTGC
*bmi1a*	GTTGATGCTGCAAATGGGTC	TTGCGCCCTATGATCGAAAA
*pcgf6*	ACTGAGAGGGCTTGAAGTTCC	CCCACAAACTCCAACATCAG
*ezh1*	ACGGCGATTTGACTGGAACA	AGGAAGCGTCTAGTGAGGTCT
*suz12b*	GTCAGCAAGAAGAGGGCTAC	CGGGTTGAGAGAGGTTTAGC
*pcgf5a*	TCTCTCCGGTTCTCACTACC	GCGCCAGCGATTTCATTATC
*pcgf5b*	AATAATGGGCAAAGACCACA	GACTTGCCATTCAATTTGCT
*rbbp4*	CACACTGCAGAGGTCAACTG	TTTCATCTTTGTGCGACTCA
*rsp18*	CATCCCAGAGAAGTTTCAGCACATC	CGCCTTCCAACACCCTTAATAGC

### Probe synthesis for whole mount *in situ* hybridization (WISH)

**Fig 1:**
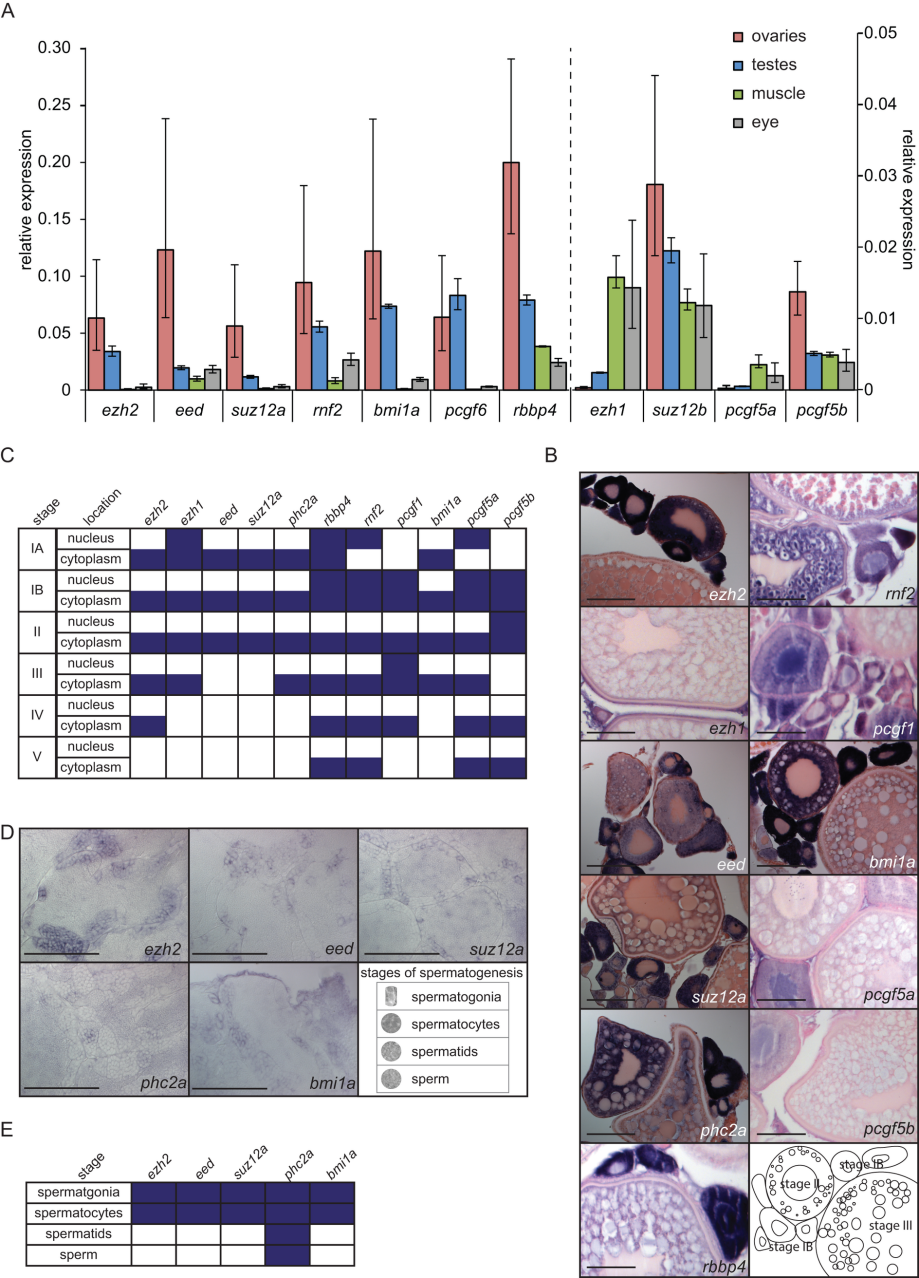
Expression of the majority of the PcG genes that were tested is enriched in the adult germ line. (A) Relative expression assessed by RT-qPCR for *ezh2*, *eed*, *suz12a*, *rnf2*, *bmi1a*, *pcgf6*, and *rbbp4* and for the lower expressed genes *ezh1*, *suz12b*, *pcgf5a*, and *pcgf5b* in the adult ovary and testis and two somatic tissues: muscle and eye. Relative expression to the reference gene *rsp18* is shown. Data based on three biological replicates and three technical replicates. Error bars indicate standard deviation. (B) Spatio-temporal expression of *ezh2*, *ehz1*, *eed*, *suz12a*, *phc2a*, *rbbp4*, *rnf2*, *pcgf1*, *bmi1a*, *pcgf5a*, *pcgf5b*, and *pcgf6* in adult ovaries. Lower right panel shows a schematic representation of stage IB, II, and III of oogenesis. Stages of oogenesis are assessed according to Selman *et al*. [32]. Scale bar: 100 μm. (C) Expression of the PcG genes that were tested in different stages of oogenesis. Purple boxes indicate expression of the corresponding gene at that stage of oogenesis. Stages of oogenesis are assessed according to Selman *et al*. [32]. (D) Spatio-temporal expression of *ezh2*, *eed*, *suz12a*, *phc2a*, and *bmi1a* in adult testes. Lower right panel shows examples of the four stages of spermatogenesis. Scale bar: 100 μm. (E) Expression of the PcG genes that were tested in different stages of spermatogenesis. Purple boxes indicate positive expression of the corresponding gene at that stage of spermatogenesis. Stages of spermatogenesis are determined according to Leal *et al*. [33].

**Fig 2:**
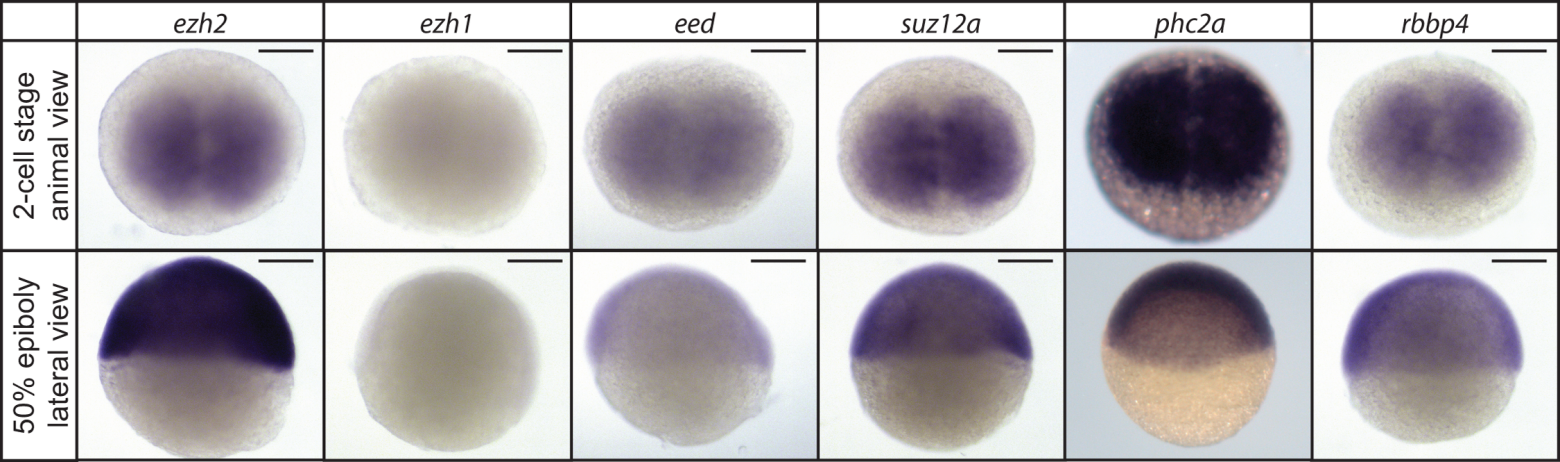
Expression of PRC2 members at early stages of embryonic development. Spatio-temporal expression assessed by whole mount *in situ* hybridization of *ezh2*, *ezh1*, *eed*, *suz12a*, *phc2a* and *rbbp4*, at the 2-cell stage (0.75 hpf) and 50% epiboly (5.3 hpf). Scale bar: 200 μm.

**Fig 3:**
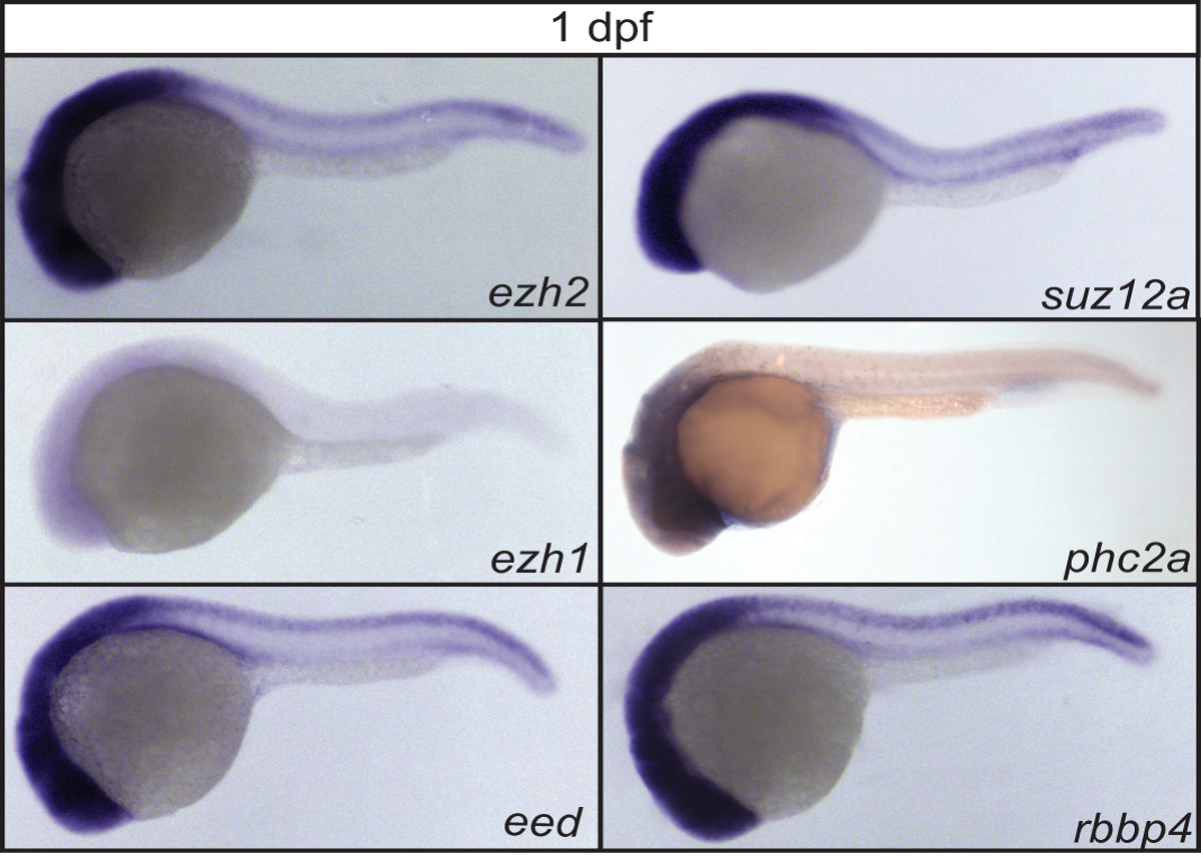
Expression of PRC2 members at 1 dpf. Lateral view of the spatio-temporal expression assessed by whole mount *in situ* hybridization of the PRC2 members: *ezh2*, *ezh1*, *eed*, *suz12a*, *phc2a*, and *rbbp4*, at 1 dpf zebrafish embryos.

**Fig 4:**
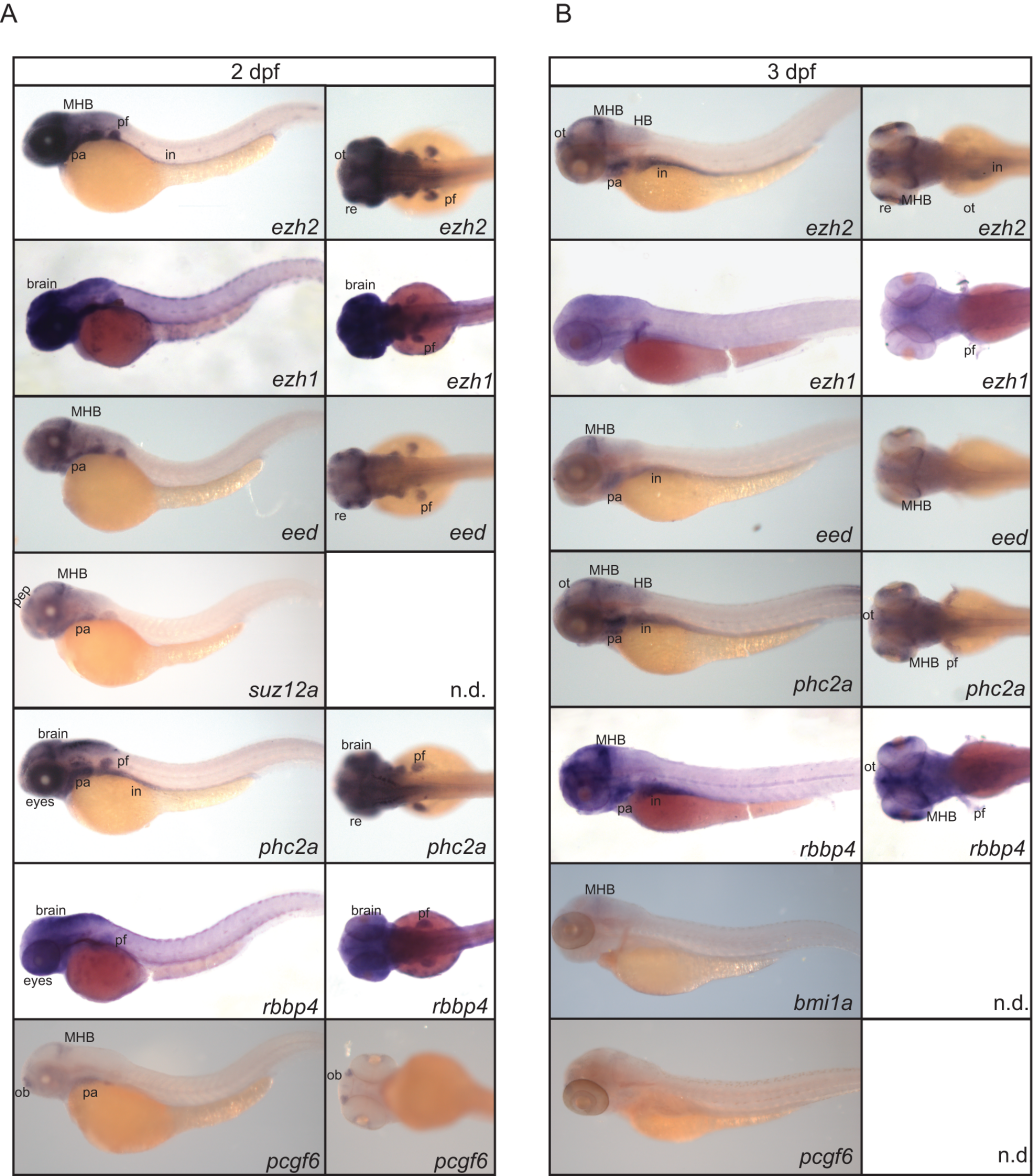
Expression of PcG genes at 2 and 3 dpf. (A) Spatio-temporal expression assessed by whole mount *in situ* hybridization of *ezh2*, *ezh1*, *eed*, *suz12a*, *phc2a*, *rbbp4*, and *pcgf6* at 2 dpf. Lateral views are shown for all genes. Dorsal views of *ezh2*, *ehz1*, *eed*, *phc2a*, and *rbbp4*. Ventral view of *pcgf6*. (B) Spatio-temporal expression assessed by whole mount *in situ* hybridization of *ezh2*, *ezh1*, *eed*, *phc2a*, *rbbp4*, *bmi1a*, and *pcgf6* at 3 dpf. Lateral views are shown for all genes. Dorsal views of *ezh2*, *ezh1*, *eed*, *phc2a*, and *rbbp4*. in: intestine, pf: pectoral fin (buds), HB: hindbrain, MHB: mid-hind brain boundary, pep: presumptive epiphysis, pa: pharyngeal arches 3–7, ot: optic tectum, re: retina, ob: olfactory bulb, n.d. = no data.

Antisense RNA probes were *in vitro* transcribed as described before [4,11]. Plasmids containing PcG genes *bmi1a*, *eed*, *suz12a*, *phc2a*, *ezh2*, and *ezh1* were ordered at Imagen and if needed subcloned in pCS2+, followed by linearization and *in vitro* transcription using T3, SP6, or T7 RNA polymerase (details in Table 2). Antisense *rbbp4* riboprobe was amplified from cDNA using a forward *rbbp4* primer 5’-CAGGCCCTTTAGTGAGGGTTAATTcacactgcagaggtcaactg-3’ with a T7 tag (T7 sequence in capitals), and a reverse primer for *rbbp4* 5’-CAGG TAATACGACTCACTATAGGG-cctgaacctcagtgtctgct-3’ tagged with a T7 polymerase sequence (T7 sequence in capitals). Template cDNA was generated by reverse-transcribing RNA which was isolated from adult whole fish tissue. After PCR clean-up T7 RNA polymerase was used to prepare the RNA probe. The probe is used for WISH shown in Figs 1B, 2, 3, and 4.

**Table 2:** Whole mount *in situ* hybridization probes from plasmids.

Gene name	Clone number	Plasmid	Accession number	restriction enzyme for linearization	polymerase for IVT	Used in Figure
*ezh2*	IRAKp961B17283Q	pME18S-FL3	AW175260, BC124588, AW170898	NotI, NheI	SP6	1, 2, 3, 4, 5
*eed*	IRBOp991D1166D	pME18S-FL3	CO935796, BC093351	KpnI, XbaI	SP6	1, 2, 3, 4, 5
*suz12a*	IRALp962D0460Q	pDNR-LIB	BC078293, CK679253	EcoRI, SpeI	T7	1, 2, 3, 4, 5
*phc2a*	IRAKp961K13101Q	pME18S-FL3	AW422704, BC044345, AW343587	BamHI	T3	1, 2, 3, 4, 5
*rnf2*	IRBOp991A1115D	pME18S-FL3	BF157635, BC044472, BG302967	HindIII	T7	1
*bmi1a*	IRBOp991C0922D	pME18S-FL3	BC049423, BM957934, BM958248	BamHI, EcoRV	T3	1, 4, 5
*pcfg6*	IRAKp961I23304Q	pExpress-1	EE326287, BC139554	EcoRI	T7	4
*ezh1*	IRBOp991F1276D	pExpress-1	CN509434, BC114282	XhoI, BamHI	SP6	1, 2, 3, 4
*pcgf1*	n.a.	SPT18	BC134012.1	NarI	T7	1
*pcgf5a*	n.a.	SPT19	BC163687.1	HindIII	T7	1
*pcgf5b*	n.a.	SPT20	BC116619.1	NarI	T7	1

IVT: *in vitro* transcription, n.a: not applicable

### Whole mount *In Situ* hybridization (WISH)

Embryos (2-cell stage or 50% epiboly) were fixed overnight at 4°C in 4% PFA (Aurion, 151710) in PBST (PBS with 0.1% Tween-20), after which they were washed with PBST, dechorionated, and gradually transferred to 100% methanol. Embryos of 1, 2, and 3 dpf were dechorionated when needed and subsequently fixed overnight at 4°C in 4% PFA in PBST, after which they were washed with PBST and gradually transferred to 100% methanol. Prior to WISH, embryos were transferred back to PBST. WISH was performed as described previously [28,29]. The embryos were mounted in methylcellulose (4%) and imaged by light microscopy on a Leica MZFLIII, with a Leica DFC450 camera or on a Leica DM2500 microscope with a Leica DFC7000T camera.

### WISH on gonads

Adult gonads were dissected from euthanized adult zebrafish [27] and fixed overnight at 4°C in 4% PFA in PBST, after which they were gradually transferred to 100% methanol in steps of 1 hour. Gonads were transferred to PBST before starting WISH. WISH on gonads was performed as described previously [28,30]. After stopping the staining reaction, gonads were stored in 100% methanol.

### Histology

After WISH, gonads (ovaries and testis) were dehydrated stepwise with ethanol and xylene. Subsequently, gonads were transferred to plastic or paraffin overnight. The following day tissues were embedded in plastic or paraffin and sectioned (5 μm for testis and 10 μm for ovaries). Gonads on which WISH for *ezh2*, *eed*, *suz12a*, *phc2a*, or *bmi1a* was performed, were embedded in plastic and ovaries on which WISH for *ezh1*, *rbbp4*, *rnf2*, *pcgf1*, *pcgf5a*, or *pcgf5b* was performed, were embedded in paraffin. For counterstaining of ovaries neutral red (Sigma-Aldrich, 0.1%) (plastic sections) or nuclear fast red (N3020, Sigma-Aldrich) (paraffin sections) was used. Slides were dewaxed (3x5 minutes xylene, 2x1 minute 100% alcohol, 2x1 min. 95% alcohol, 1 min. 70% alcohol, 1 min. 50% alcohol, 1 minute H_2_O) and incubated for 10 minutes in 0.1% neutral red or 0.1% nuclear fast red [30,31]. Sections were washed briefly with water, dehydrated with xylene, and covered with a cover slip using permount or Depex for future imaging. Images of plastic embedded tissue were made using a Zeiss Axioplan microscope equipped with a Zeiss Axiocam digital camera. Paraffin sections were imaged using a Leica DM2500 microscope with a Leica DFC7000T camera.

## Results

### PcG expression is enriched in the adult germ cells over somatic tissue

The expression level of the PcG genes *ezh2*, *ezh1*, *eed*, *suz12a*, *suz12b*, *rbbp4*, *bmi1a*, *pcgf5a*, *pcgf5b*, *pcgf6*, *and rnf2* was assessed by RT-qPCR from adult male and female gonads and two somatic tissues: muscle and eye. The expression of the reference gene *rsp18* was used for normalization. Enrichment in gene expression of *ezh2*, *eed*, *suz12a*, *rnf2*, *bmi1a*, *pcgf6*, *rbbp4*, *suz12b*, and *pcgf5b* was detected in the germ line over somatic tissue (Fig 1A, S1 Table). As a next step, we investigated the expression of a selection of PcG genes in adult germ cells in more detail using whole mount *in situ* hybridization (WISH, Fig 1B–1E).

Oogenesis encompasses 6 different stages of oocytes [32]. Assessment of the different stages was performed based on morphology [32]. Eleven different PcG genes were tested in the adult ovary (Fig 1B). mRNA expression in the cytoplasm and nucleus throughout the different stages of oogenesis is summarized in Fig 1C. In stage IA oocytes, which are cells in the pre-follicle phase, we detected *ezh2*, *ezh1*, *eed*, *suz12a*, *phc2a*, *rbbp4*, and *bmi1a* in the cytoplasm. Only *ezh1*, *rbbp4*, *rnf2*, and *pcgf5a* were detected in the nucleus at stage IA. In stage IB oocytes the expression of *rbbp4*, *rnf2*, *pcgf1*, *pcgf5a*, and *pcgf5b* was detected in both the nucleus and cytoplasm. The expression of *ezh2*, *ezh1*, *eed*, *suz12a*, *phc2a*, and *bmi1a* was only visible in the cytoplasm. Stage II oocytes showed expression of all PcG genes tested in their cytoplasm. Additionally, *pcgf5b* was also detected in the nuclei of stage II oocytes. In stage III-V expression of the majority of the PcG genes we tested is not detected in the nucleus. Only *pcgf1* mRNA was detected in stage III oocytes. The PcG genes *ezh2*, *ezh1*, *phc2a*, *rbbp4*, *rnf2*, *pcgf1*, *bmi1a*, and *pcgf5a* show expression in the cytoplasm of stage III oocytes. In stage IV expression of *ezh2*, *rbbp4*, *rnf2*, *pcgf1*, *pcgf5a*, and *pcgf5b* was detected in the cytoplasm. Lastly, *rbbp4*, *rnf2*, *pcgf5a*, and *pcgf5b* expression was detected in the cytoplasm of stage V oocytes.

Additionally, we tested the expression of *ezh2*, *eed*, *suz12a*, *phc2a*, and *bmi1a* in adult testes. We discriminated between four different stages of spermatogenesis according to the morphology of the cells, as described by Leal *et al*. [33] Early stages of spermatogenesis, the spermatogonia and spermatids showed expression of *ezh2*, *eed*, *suz12a*, *phc2a*, and *bmi1a* (Fig 1D and 1E). Mature stages of spermatogenesis: spermatids and sperm showed expression of *phc2a* (Fig 1D and 1E).

### The majority of PRC2 genes is maternally provided

To determine whether components of PRC2 are maternally provided and therefore present before zygotic genome activation (ZGA) we performed whole mount *in situ* hybridization on 2-cell stage embryos (0.75 hpf; Fig 2). We observed that *ezh2*, *eed*, *suz12a*, *phc2a* and *rbbp4*, are maternally provided to the embryo, but there was no detectable presence of *ezh1* mRNA. In addition, we also studied the gene expression of these PRC2 components after ZGA, at 50% epiboly, and observed ubiquitous expression for *ezh2*, *eed*, *suz12a*, *phc2a*, and *rbbp4* (5.3 hpf; Fig 2). We did not detect expression of *ezh1* at 50% epiboly.

### Expression of a selection of PcG genes during tissue specification

To investigate the expression patterns of a selection of PcG genes during tissue specification, we next performed whole mount *in situ* hybridization at 1, 2, and 3 dpf. Whereas the expression of the majority of the PcG genes we tested, except for *ezh1*, was ubiquitously present at early stages (2-cell and 50% epiboly), the expression becomes more anteriorly enriched at 1 dpf (Fig 3). The expression of *ezh2*, *eed*, *suz12a*, *phc2a*, and *rbbp4* was clearly visible. However, mRNA expression of *ezh1* was detected at a very low level at the anterior side of the embryo.

Spatial restriction of the expression of this selection of PcG genes becomes more apparent at 2 and 3 dpf (Fig 4). At 2 dpf the transcripts of *ezh2*, *ezh1*, *eed*, *suz12a*, *phc2a*, *rbbp4*, and *pcgf6* were enriched in brain tissue, such as the mid-hindbrain barrier, and expressed in the pharyncheal arches (Fig 4A). Furthermore, *ezh2*, *ezh1*, *eed*, *rbbp4*, and *phc2a* are expressed in the pectoral fin buds and both *ezh2* and *phc2a* were detected in the intestine at 2 dpf (Fig 4A). The PRC2-components *ezh2*, *eed*, *rbbp4*, and *phc2a* are expressed in the intestine at 3 dpf. At 3 dpf *ezh2*, *eed*, *phc2a*, *rbbp4*, and *bmi1a* were expressed in the mid-hindbrain barrier. Overall, enrichment of anterior expression was observed for *ezh2*, *eed*, *phc2a*, and *rbbp4* at 3 dpf (Fig 4B).

### The expression of a selection of PcG genes during germ cell development is detectable as from 4 weeks post fertilization

**Fig 5:**
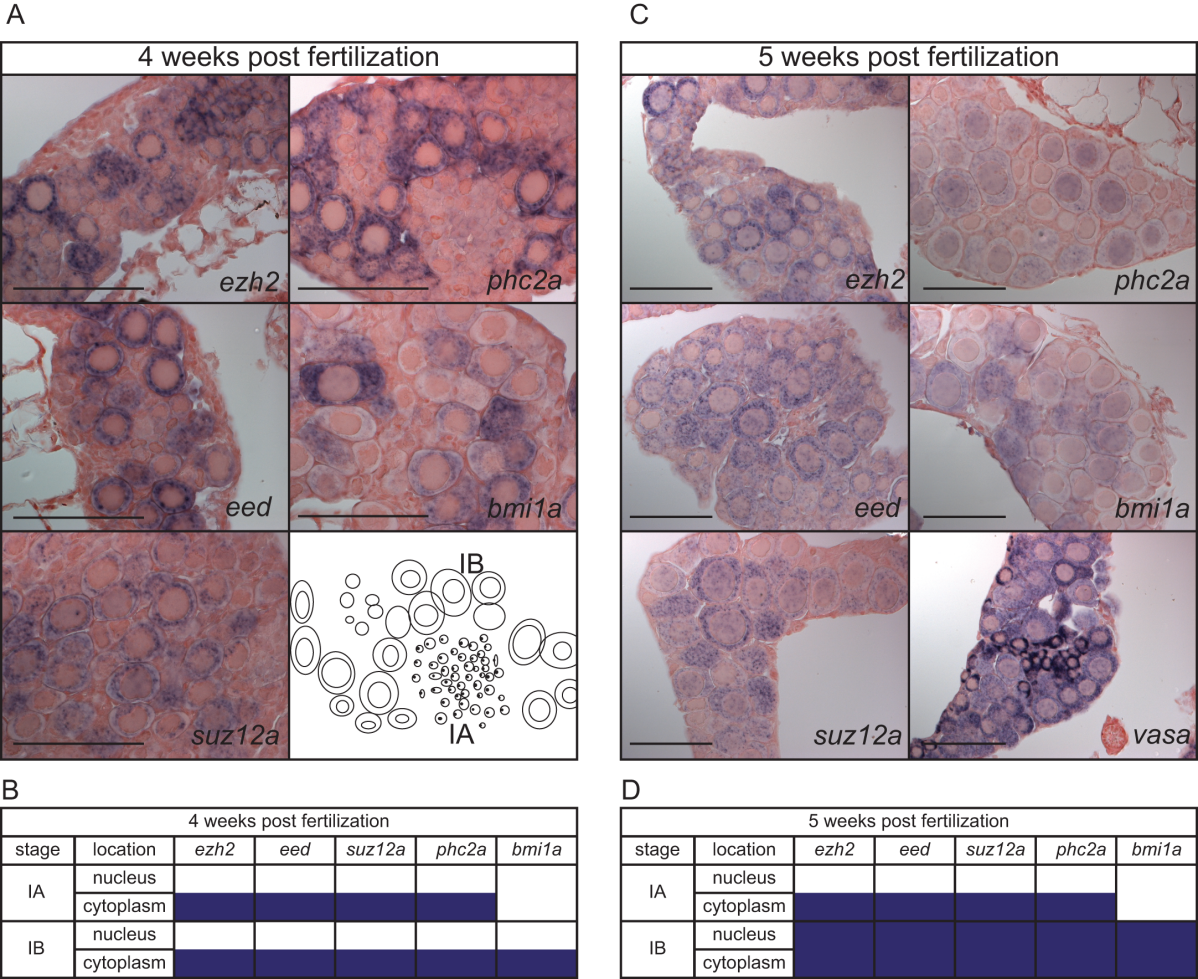
Expression of PcG genes in developing gonads. (A) Spatio-temporal expression assessed by whole mount *in situ* hybridization of *ezh2*, *eed*, *suz12a*, *phc2a*, and *bmi1a* at 4 weeks post fertilization. (B) Expression patterns of *ezh2*, *eed*, *suz12a*, *phc2a*, and *bmi1a*, in gonads at 4 weeks post fertilization. Purple boxes indicate expression of the corresponding gene at that stage of oogenesis. (C) Spatio-temporal expression assessed by whole mount *in situ* hybridization at 5 weeks post fertilization of *ezh2*, *eed*, *suz12a*, *phc2a*, *bmi1a*, and the germ cell marker *vasa*. (D). Expression of *ezh2*, *eed*, *suz12a*, *phc2a*, and *bmi1a*, at 5 weeks post fertilization. Purple boxes indicate expression of the corresponding gene at that stage of oogenesis. Stages of oogenesis are assessed according to Selman *et al*. [32].

In adult gametes expression of the tested PcG genes was detected, but we did not observe them in primordial germ cells at 1, 2, or 3 dpf (Figs 3 and 4). To determine when these PcG genes start to be expressed in the developing germ line, we performed whole mount *in situ* hybridization at gonads from 3 to 5 weeks post fertilization for a subset of PcG genes. We were not able to detect the expression of these PcG genes at 3 weeks post fertilization in the developing gonads. At 4 weeks post fertilization we detected the expression of *ezh2*, *eed*, *suz12a*, and *phc2a* in the cytoplasm of stage IA oocytes. Furthermore, all genes tested were detected in the cytoplasm of stage IB oocytes (Fig 5A and 5B). We additionally tested expression of *ezh2*, *eed*, *suz12a*, *phc2a*, and *bmi1a*, in presumptive ovaries at 5 weeks post fertilization, and expression was observed for all, except *bmi1a*, in the cytoplasm of the stage IA oocytes. At 5 weeks post fertilization, all PcG genes tested were detected in both the nucleus and the cytoplasm of stage IB oocytes. As a control we used *vasa*, a germ cell marker (Fig 5C).

## Discussion

### PcG genes are expressed in the germ line of adult zebrafish

Safeguarding germ cell fate is extremely important since germ cells form the basis for the existence and propagation of a species. Therefore, correct gene expression must be maintained in germ cells. Amongst the mechanisms that are reported for this maintenance are transcriptional repression, chromatin state protection, and protection of genome integrity [34]. Transcriptional repression is described to be an important event and therefore investigating the expression of PcG genes in adult gametes as well as in developing gonads will give us insight in the potential role of PcG-mediated gene repression in zebrafish germ cells. In this study we found that expression of nine out of eleven PcG genes that were tested is enriched in the germ line over somatic tissue in adult zebrafish. The mRNA levels of *ezh1* and *pcgf5a* are higher in eye and muscle tissue, compared to gonad tissue. Zygotes are reported to not have maternal load of *ezh1* and *pcgf5a* [24]. The lack of maternal load for these genes could be a correlated to our observation of low expression of these genes in adult gonads [24].

Because germ cells have the capacity to form a totipotent zygote upon fertilization, it is expected that germ cells remain in a relatively quiescent state compared to somatic cells [34]. Germ cells can be regarded as a stem cell-like model. They both have the potential to give rise to all different cell types. PcG gene expression is associated to stem cell maintenance [35]. Detailed spatio-temporal expression analysis of eleven different PcG genes in ovaries indicates that stage IB oocytes show mRNA expression of PRC1 members in both the nucleus and the cytoplasm which is in contrast to the mature stages of the oogenesis, where only *rbbp4*, *rnf2*, *pcgf5a*, and *pcgf5b* mRNAs are detected in the cytoplasm. The other genes tested were not detected in stage V oocytes. PcG proteins act as transcriptional repressors and since we mainly observe their expression in stage I-II oocytes, we hypothesize that transcription is actively repressed by PcG proteins in early stage oocytes. These early stage oocytes can be considered as the germ line stem cells. The presence of PcG genes in these early stages of oogenesis suggests that especially these cells undergo chromatin remodeling and changes in the epigenome.

In later stages of oogenesis we did not detect the mRNA of the tested PcG genes in the nucleus. In these mature oocytes we are often also not able to detect PcG gene expression in the cytoplasm. One can regard the content of the cytoplasm as the maternal load that is transmitted to the zygote. We analyzed the expression of the PRC2 members by whole mount *in situ* hybridization at the 2-cell stage. We found *ezh2*, *eed*, *suz12a*, *phc2a*, and *rbbp4* to be present, and did not detect *ezh1*. Since zygotic genome activation occurs after the 2-cell stage, this means that the PRC2 members tested, except *ezh1*, are maternally loaded. The RT-qPCR results also indicate that *ezh1* is expressed at low levels in the gonads. The transcripts of PRC1 components *rnf2*, *pcgf1*, *bmi1a*, *bmi1b*, *pcgf5a*, and *pcgf6* were also reported to be maternally loaded [4,13]. The PRC1 component *pcgf5b* was reported not to be present at the 2-cell stage [4]. Stage V oocytes are the basis for the zygote and therefore the PcG transcripts that are maternally provided are also expected to be present in the cytoplasm of stage V oocytes. However, this is not what we consistently observed. A potential explanation for why *ezh2*, *eed*, *suz12a*, *phc2a*, *pcgf1*, and *bmi1a* are not observed in stage V oocytes could be that the mRNA has been diluted, and is therefore not detectable by WISH, in these large stage V oocytes.

Ezh2 is maternally loaded and we find this mRNA also to be present in the germ line. Likely, during germ line development, Ezh2 plays a role in regulating transcription. Therefore, it was unexpected that zebrafish with an *ezh2* mutant germ line are fertile [11]. Ezh2 inhibitors are sometimes used as anti-cancer drugs and pharmacological inhibition of Ezh2 in the murine germ line resulted in depletion of H3K27me3 [18,36]. What the effects are on fertility is an important remaining question.

The testis, especially the early stages of spermatogenesis, the spermatogonia and spermatocytes, showed expression of *ezh2*, *eed*, *suz12a*, *phc2a*, and *bmi1a*. The analogy of presence of PcG genes in the early stages of gametogenesis, in both the female and male germ line, hints towards a conserved mechanism that requires PcG protein functioning in early gametogenesis.

### Role of PcG genes in embryogenesis

Once the stage V oocyte is fertilized, a zygote develops with maternal load consisting of a number of mRNA transcripts. Based on published whole mount *in situ* hybridization experiments, PRC1 members *rnf2* and *pcgf1-6*, except *pcgf5b*, are maternally loaded [4,13]. This contradicts the results of *pcgf5a* and *pcgf5b* expression during development as was found by a high-resolution mRNA expression time course of embryonic development in zebrafish [24]. The expression of *pcgf5a* is reported to be absent until 75% epiboly and expression is first observed at the 1–4 somite [24]. Before ZGA expression of *pcgf5b* is already observed, when analyzed by RNA-seq. A potential explanation for this finding is the high similarity between these two transcripts, which could make distinguishing them problematic, both by RNA-sequencing as well as by whole mount *in situ* hybridization.

At early stages of embryogenesis the PRC2 members were ubiquitously expressed, except *ezh1*, which was not detected. The expression of *ezh2* is similar to previously published [11]. The majority of the genes that we tested by whole mount *in situ* hybridization at 1, 2, and 3 dpf are detected in the brain regions, especially in the mid-hindbrain barrier. The pattern of PcG expression presented here shows high resemblance with the expression of the proliferation marker *pcna* [37]. PcG genes are described to enhance proliferation and the overlap in expression patterns found with the pattern of *pcna* could suggest that PcG genes are expressed in regions that are highly proliferative [38].

Some PcG genes are duplicated in the zebrafish genome, this includes the previously discussed *pcgf5* gene [5]. Another example is the PRC1 component *bmi1;* the two paralogues were reported to have different expression patterns [4]. The *phc2* gene is also duplicated. Additionally, there are two isoforms of *phc2a* described, which adds an extra layer of complexity to studying these type of genes [6]. The *phc2a* probe we have used for our analysis does not distinguish between these isoforms. The different paralogues of the PcG genes and the possibility of different isoforms should be taken into account when studying these genes in zebrafish.

The data presented in this study can contribute to our understanding of explaining the phenotypes of PcG gene mutants in zebrafish reported so far. For instance, we detect *ezh2* expression in the pectoral fin buds, brain region, especially at the mid-hind brain barrier, and the intestinal tract. The *MZezh2* mutants lack pectoral fins, and show malformation of the head, which could indicate a need for Ezh2 for proper outgrow of these tissues [11]. Normally during embryonic development, the gut is formed around 5 dpf. In this study, we could detect expression of *ezh2* at 2 and 3 dpf in the presumptive gut tissue. Additionally, Dupret *et al*. reported intestinal defects in zygotic *ezh2* mutant zebrafish [12]. PRC1 was also implicated to play a role in pectoral fin development, as zygotic *rnf2* mutants show defects in fin bud outgrowth, due to incomplete activation of the Fgf signaling pathway. In these mutants, initial specification from presumptive pectoral fin precursors is correctly initiated. This observation confirms the view that PcG genes are involved in terminal differentiation of different tissue types [39]. Since *eed* and *phc2a* expression is detected in the pectoral fin buds at 2 dpf, we hypothesize that *eed* and *phc2a* zebrafish mutants would also lack pectoral fins.

### The enzymatic subunit of PRC2 during zebrafish development

PRC2 contains one enzymatic subunit, Ezh1 or Ezh2, which are mutually exclusive [40,41]. The expression pattern of *ezh1* is visually different from the other PRC2 components, since *ezh1* seems to be the only PRC2 member which is not maternally loaded. Additionally, *ezh1* cannot be detected at 2-cell stage and does not seem to be expressed up until 50% epiboly. This suggests that this gene is not activated at ZGA. A similar observation was made by Sun *et al*.; the expression of *ezh2* was detected at 0.75 hpf (2-cell stage), 2 hpf, and 4 hpf, but no expression of *ezh1* was observed at these stages [25]. At 1 dpf we observed *ezh1* expression at low levels. Similar data about expression of *ezh1* were also found in a high-resolution mRNA expression time course of embryonic development in zebrafish [24]. WISH at 2 and 3 dpf for *ezh1* indicates an expression pattern that is enriched in the head region and the pectoral fins. At 5 dpf *ezh1* expression is clearly detected in the RNA-sequencing dataset [24]. The expression levels in the study of White *et al*. show that at 1 dpf the levels are roughly 47 times higher for *ezh2* compared to *ezh1* [24]. The current view on Ezh1/Ezh2 is that Ezh2 is incorporated in PRC2 in cells that are pluripotent, such as embryonic stem cells. Ezh1 is believed to be present in adult tissue and non-proliferative cells [40,41]. Our observation of enrichment of *ezh1* expression in muscle and eye tissue over gonad tissue is in line with this view. The cells of a 2-cell stage embryo have the potential to grow into all different cell types, which resembles a stem-cell like state, and this could explain why *ezh2* is maternally loaded and *ezh1* is not. Only at later stages of development *ezh1* expression is detected. These observations support the hypothesis that Ezh2 and Ezh1 are part of PRC2 in less or more differentiated cells, respectively.

### Summary and future perspectives

In this study we have shown the expression of a selection of PcG genes in the germ line and during early embryonic development. A multitude of the PcG genes that we tested showed enrichment in the germ line over somatic tissue. We also found that especially early stages of gametogenesis showed expression of the PcG genes that we tested. During zebrafish embryonic development the majority of PcG genes were also detected, this is a ubiquitous expression in the early stages and becomes more anteriorly enriched from 1 dpf onwards. The observations described here underline the likelihood for a role of PcG genes in specification of multiple lineages, including the germ line. Mutants are pivotal to study the function of a gene or multiple genes during development. Zebrafish are more frequently used to study epigenetics and some PcG mutants are already generated in the Sanger Institute and available via Zebrafish International Resourc Center (ZIRC). Nowadays, new zebrafish mutants are relatively easily generated using the CRISPR-Cas9 system [42]. This system allows for targeted mutations and provides opportunities to further study the role and function of PcG proteins in zebrafish embryonic and adult development. One could make mutants for the different paralogues of PcG genes and aim to mutate the different isoforms. Our results serve as an important resource of information on the expression patterns of a selection of PcG genes during embryonic and germ line development. This contributes to our understanding of the role of the PcG proteins, which were tested here, in embryogenesis and germ line development. Follow-up studies need to be performed in order to obtain detailed insights.

## Supporting information

S1 Table Ct values of RT-qPCR performed for a selection of PcG genes on different adult tissues. (XLSX)
